# Using full chloroplast genomes of ‘red’ and ‘yellow’ *Bixa orellana* (achiote) for kmer based identification and phylogenetic inference

**DOI:** 10.1186/s12864-020-06916-0

**Published:** 2020-08-06

**Authors:** Jorge Villacrés-Vallejo, José Aranda-Ventura, Anna Wallis, Robin Cagle, Sara M. Handy, Jeffery Davis, Elizabeth Reed, Shu Zhang, Errol Strain, Monica Pava-Ripoll, David Erickson, Padmini Ramachandran, Andrea Ottesen

**Affiliations:** 1 Seguro Social de Salud, Instituto de Medicina Tradicional, Iquitos, Peru; 2 Universidad Nacional de la Amazonía Peruana, Facultad de Agronomía, Iquitos, Peru; 3 Department of Plant Pathology, Cornell University, Ithaca, NY USA; 4 Office of Regulatory Science, Center for Food Safety and Applied Nutrition, FDA, College Park, MD USA; 5 Department of Chemistry and Biochemistry, University of Maryland, College Park, USA; 6 DNA4 Technologies LLC, Baltimore, MD USA; 7 Office of Research, Center for Veterinary Medicine, FDA, Laurel, MD USA; 8 Office of Food Safety, Center for Food Safety and Applied Nutrition, FDA, College Park, MD USA; 9 Joint Institute for Food Safety and Applied Nutrition, College Park, MD USA; 10 Department of Plant Sciences and Landscape Architecture, University of Maryland, College Park, MD USA

**Keywords:** *Bixa orellana*, Achiote, Annatto, Achote, Red and yellow achiote, Bixin, Norbixin, Food coloring, Bixaceae, Malvales, Kmer, Next generation sequencing (NGS), Center for Veterinary Medicine (CVM) complete chloroplast animal feed database, National Antimicrobial Resistance Monitoring System (NARMS), Instituto de Medicina Tradicional (IMET), Iquitos, Peru

## Abstract

**Background:**

Full chloroplast genomes provide high resolution taxonomic discrimination between closely related plant species and are quickly replacing single and multi-locus barcoding regions as reference materials of choice for DNA based taxonomic annotation of plants. *Bixa orellana*, commonly known as “achiote” and “annatto” is a plant used for both human and animal foods and was thus identified for full chloroplast sequencing for the Center for Veterinary Medicine (CVM) Complete Chloroplast Animal Feed database. This work was conducted in collaboration with the Instituto de Medicina Tradicional (IMET) in Iquitos, Peru. There is a wide range of color variation in pods of *Bixa orellana* for which genetic loci that distinguish phenotypes have not yet been identified. Here we apply whole chloroplast genome sequencing of “red” and “yellow” individuals of *Bixa orellana* to provide high quality reference genomes to support kmer database development for use identifying this plant from complex mixtures using shotgun data. Additionally, we describe chloroplast gene content, synteny and phylogeny, and identify an indel and snp that may be associated with seed pod color.

**Results:**

Fully assembled chloroplast genomes were produced for both red and yellow *Bixa orellana* accessions (158,918 and 158,823 bp respectively). Synteny and gene content was identical to the only other previously reported full chloroplast genome of *Bixa orellana* (NC_041550). We observed a 17 base pair deletion at position 58,399–58,415 in both accessions, relative to NC_041550 and a 6 bp deletion at position 75,531–75,526 and a snp at position 86,493 in red *Bixa orellana*.

**Conclusions:**

Our data provide high quality reference genomes of individuals of red and yellow *Bixa orellana* to support kmer based identity markers for use with shotgun sequencing approaches for rapid, precise identification of *Bixa orellana* from complex mixtures. Kmer based phylogeny of full chloroplast genomes supports monophylly of Bixaceae consistent with alignment based approaches. A potentially discriminatory indel and snp were identified that may be correlated with the red phenotype.

## Background

Complete chloroplast genomes provide numerous single nucleotide polymorphisms (snps) that can often discriminate between closely related plant species [1] and even identify biogeographic diversification in the same species [2]. Thus, complete chloroplasts are quickly replacing single and multi-locus barcoding regions as reference materials of choice for DNA based taxonomic annotation of plants. The U.S. FDA has created a database of full chloroplast genomes of plants found in foods and dietary supplements (Genometrakr CP) [3], and is beginning the assembly of a concordant database focused primarily on plants used in animal feed, which will include toxin producers, common contaminants and their close relatives; the Center for Veterinary Medicine (CVM) Complete Chloroplast Animal Feed database. This work is being conducted in collaboration with the Instituto de Medicina Tradicional (IMET) in Iquitos, Peru. This repository will be also be used by the National Antimicrobial Resistance Monitoring System (NARMS) to complement genomic source tracking and epidemiology of antimicrobial resistance in pathogens and associated bacteria in animal feed ingredients and mixtures. The neotropical plant *Bixa orellana*, also known as “annatto” (English), ‘achiote’, ‘achiote amarrillo’, ‘achote’ (Spanish) ‘rocou’ (French), ‘urucu’(Brazilian Portuguese), ‘orlean’ (German), and Mzingifuri (Swahili)) [4] is widely used in human and animal food production as a food colorant and has been identified for inclusion in this database.

Effective means of identifying/authenticating *Bixa* (and all plants used in food and animal feed) is valuable for many reasons including; quality metrics, identification of potential contaminants, pathogen source tracking, description of potential antimicrobial resistance, allergens, and pesticide residues, source tracking of ingredients, etc. Recently, the use of whole chloroplast genomes as references (as opposed to DNA barcodes) [1, 3, 5–7] for plant species identification programs has provided more comprehensive utility by providing increased numbers of markers that can be applied to identification and authentication protocols [8, 9]. Identification and development of assays that confirm the identify of natural products and complex mixtures, rely on well curated databases of multiple high quality accessions [3]. With high quality references, shotgun sequencing and metagenomic approaches can provide highly accurate environmental fingerprints that include plants, bacteria, fungi, pollen, insects and mammals that may have been part of a growing, farming or production continuum for human and animal food. Source tracking endeavors for the National Antimicrobial Resistance Monitoring System (NARMS) rely on full genome sequences of pathogens and plasmids. There have been times when the closest reported pathogen genomes associated with animal products were linked to fresh produce grown for human consumption. Thus, the integration of a database of eukaryotic (plant based) ingredients and associated bacteria will increase the epidemiologic capacity of surveillance of conditions, matrices and/or vehicles of pathogens and antimicrobial resistance in both human and animal foods.

**Fig. 1:**
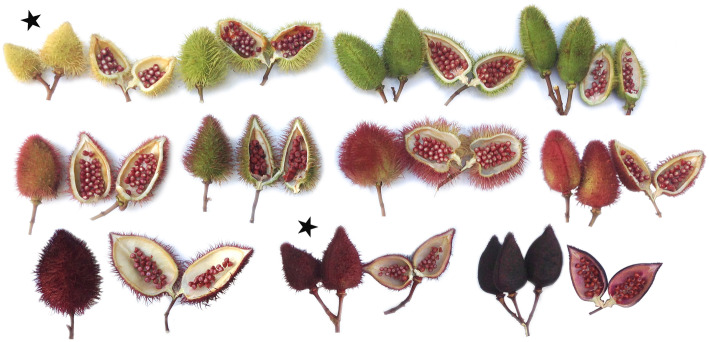
Variation in seed pod color of *Bixa orellana*. Stars represent the two samples from the germplasm collection of *Bixa orellana* from the Facultad de Agronomía - Universidad Nacional de la Amazonía Peruana (UNAP) and the Instituto de Medicina Traditional (IMET), that were used for full chloroplast sequencing

Whole chloroplast genomes also have demonstrated utility for phylogenetic reconstruction and inference of broad scale evolutionary relationships. An interesting phenomenon in *Bixa orellana* is the occurrence of multiple variations of seed pod color (from yellow to green to magenta) (Fig. 1) [10]. To identify genetic loci that distinguish color variation in seed pods, nuclear DNA may be more discriminatory, however full chloroplasts have demonstrated utility distinguishing not only closely related species but also biogeographic diversification of same species. Thus, we hypothesized that an added value to the full chloroplast sequencing for reference materials, might be - the identification of putative regions that differentiate seed pod color variants. For this reason, we selected accessioned individuals with the most diverse seed pod colors, yellow and magenta (‘red’) (Fig. 1) from the germplasm collection of the Facultad de Agronomía - Universidad Nacional de la Amazonía Peruana (UNAP), Iquitos and the Instituto de Medicina Tradicional de ES SALUD (IMET) Iquitos, Peru for full chloroplast sequencing.

Here, we compare the assembled chloroplast genomes of ‘red’ and ‘yellow’ *Bixa orellana* individuals with the only other existing published *Bixa orellana* full chloroplast accession, comparing gene count, organization and synteny. A phylogeny based on alignment free kmer distance metrics is compared to phylogenies using traditional alignment based methods with barcoding regions *matK* and *rbcL* [11] and a five gene MLST approach to explore monophylly of Bixaceae. In addition to utility for phylogenic questions and development of identity markers, we demonstrate that chloroplast genomes can be used in conjunction with modern bioinformatic search tools (kmer based) to provide rapid and precise identification of *Bixa orellana* (or any other plant) in modernized Next Generation Sequencing (NGS) approaches to identification and authentication of ingredients in complex mixtures such as natural products and animal feeds.

## Results

### Sequence assembly

**Fig. 2:**
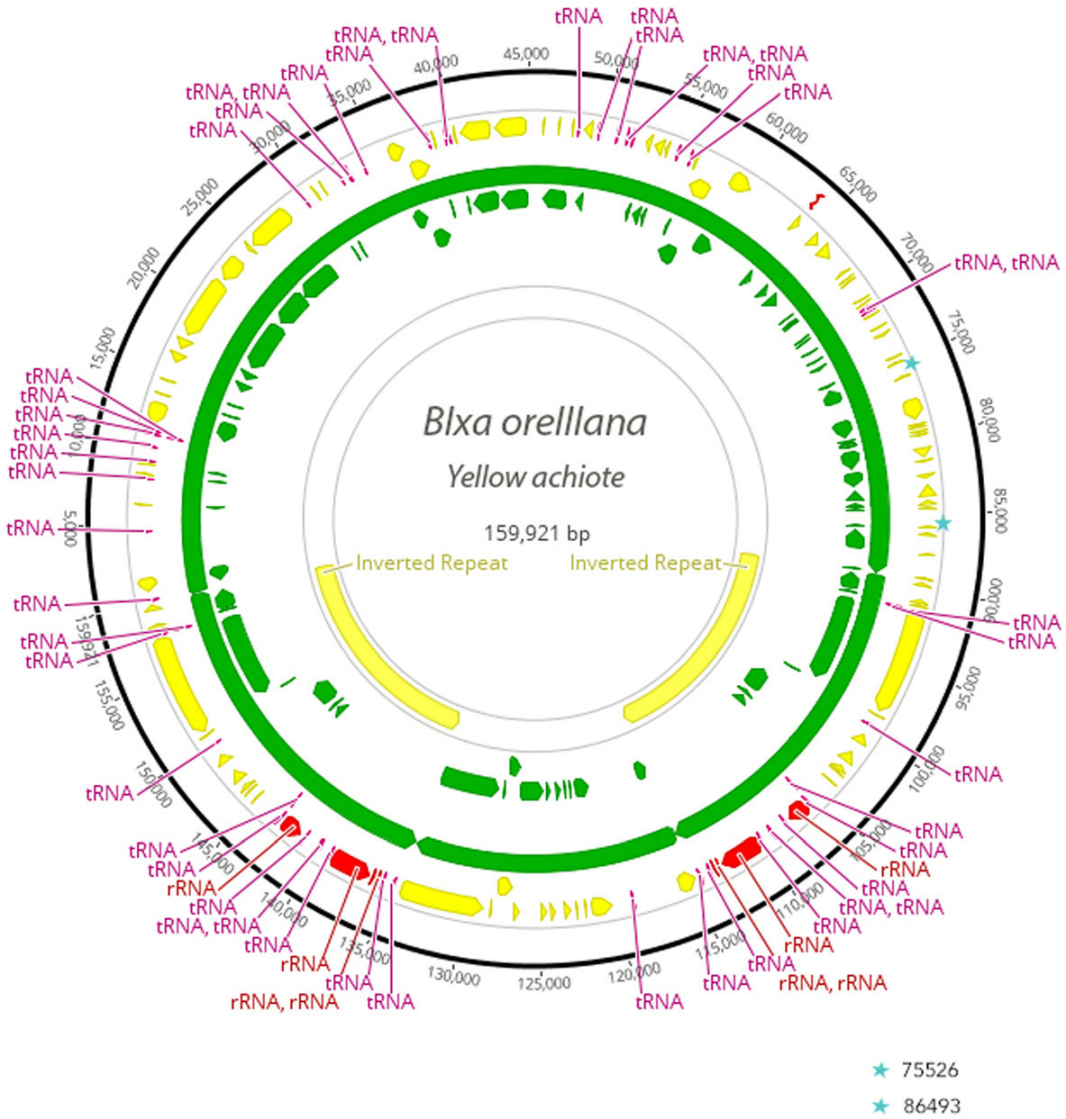
Chloroplast genomes were annotated with the Verdant online chloroplast assembly tool (http://verdant.iplantcollaborative.org/plastidDB/). The existing reference for *Bixa*, NC_041550, was added to the Verdant database prior to upload and analysis of our sequences. The resulting gff files were downloaded and used to annotate the assembled *Bixa* chloroplast genomes. Stars indicate the 6 bp deletion at bp 75,526 relative to yellow *Bixa* and a snp at bp 86,493 of ‘red’ *Bixa*

An assembled chloroplast genome was produced for both yellow and red *Bixa orellana* accessions (Fig. 2). The assembled size of the chloroplast was 158,823 and 158,918 for the yellow and red accessions respectively (Table 1). The synteny and gene content of the two accessions reported here was identical to that of NC_041550. The three *Bixa* accessions contained 129 total genes, with 85 protein coding genes, 36 tRNA and 8 rRNA (Table 1 & Table S2). The 129 genes in *Bixa* represented a gain of four genes relative to *Theobroma*, due to rpl2 being captured (and therefore duplicated) by the Inverted Repeat in *Bixa*, as well as gain of rpl22, trnG-GCC in the LSC, and ycf15 in the Inverted Repeat. *Bixa* did not have the transfer-rna trnG-UCC, which was present in *Theobroma*, resulting in a net gain of 4 genes in *Bixa* relative to *Theobroma cacao*. The overall synteny of *Theobroma* and *Bixa* was continuous, and contrasted to *Heritiera fomes*, exhibited a much smaller inverted repeat (25,000 for *Bixa* and *Theobroma* contrasted to 34,494 for *Heritiera fomes*) with an accompanying decrease in gene number (129 and 125 for *Bixa* and *Theobroma* versus 130 for *Heritiera*) due to duplication of genes in single copy portions of the genome. The representative Diperocarpaceae genome was more similar in gene structure and content to *Bixa* and *Theobroma* than was the *Heritiera*, with a similar size of the Inverted Repeat (23,911) gene number (130) and relative number of CDS, tRNA and rRNA (86, 36 and 8) (Table 1).

**Table 1:**
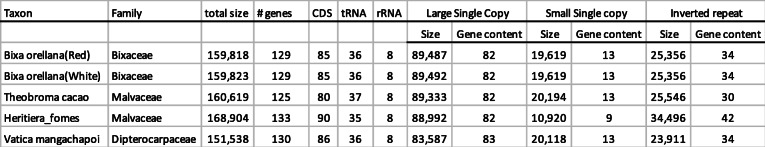
Gene content and organization.

### Sequence variation and genetic markers

MAFFT sequence alignment of the three chloroplast genomes of *Bixa* allowed us to screen the samples for genetic variants. We observed a 17 base pair deletion at position 58,399–58,415 in yellow *Bixa* FDAWG01 relative to NC_041550 that was present in both of our accessions, a 6 base pair deletion at position 75,531–75,526 and a snp at position 86,493 in ‘red’ *Bixa* FDARB01.

### Phylogenetic relationships

**Fig. 3:**
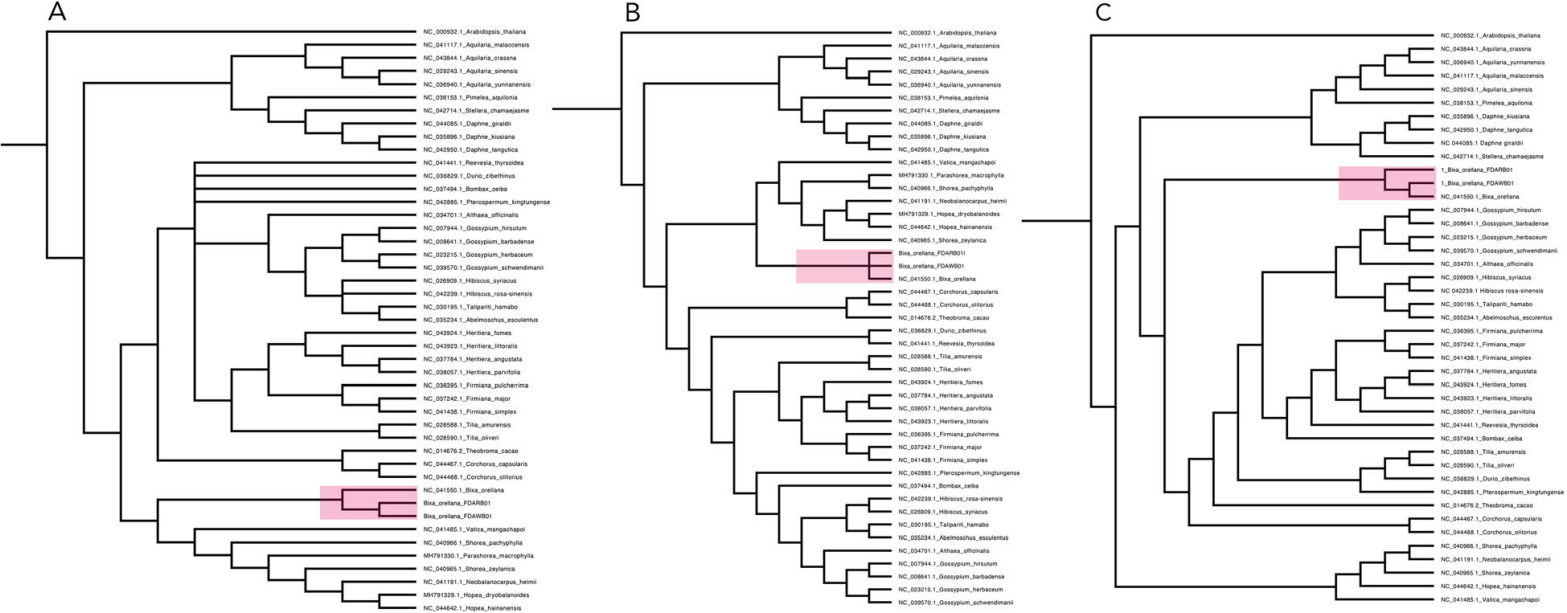
Cladograms of phylogenetic trees from: A) bar coding regions, B) five genes, and C) k-mer based phylogeny of *Bixa orellana* and other Malvales - with *Arabidopsis* as an outgroup. All approaches place Bixaceae sister to Malvales. The aim of this comparison is to demonstrate that the kmer approaches to phylogenic placement provide similar and enhanced utility to traditional universal alignment-based approaches

The use of alignment free kmer distance metrics allows for the comparison of all DNA sequence data among distantly related chloroplast genomes, not just among genes which can be easily compared through sequence alignment. To demonstrate that organization using a kmer approach is consistent with traditional alignment based approaches, we contrast kmer phylograms with those of barcoding regions matK and rbcL and a 5 gene MLST. With the kmer approach, we were able to make direct comparisons among the full genomes of relatively divergent species (4 families of Malvales and one Brassicales out group) (Fig. 3). The D2 metric distance matrix (Table S3) produced a NJ tree that places Bixaceae sister to Malvaceae within the Malvales (similar to that observed by Pacheco et al. (2019)) [10]. The three *Bixa* accessions grouped together in a discrete monophyletic group no matter which approach was used, reflecting the accuracy of the kmer assembly, and the putative value of the entire chloroplast as a diagnostic marker for the group. The Thymelaeaceae were sister to the Bixaceae-Malvaceae clade, with the Dipterocarpaceae basal within the order. This differs to the APG IV assessment (www.mobot.org/MOBOT/research/APweb/) which places Thymelaeaceae basal to both Dipterocarpaceae and Bixaceae.

### Kmer based applications for identification

**Fig. 4:**
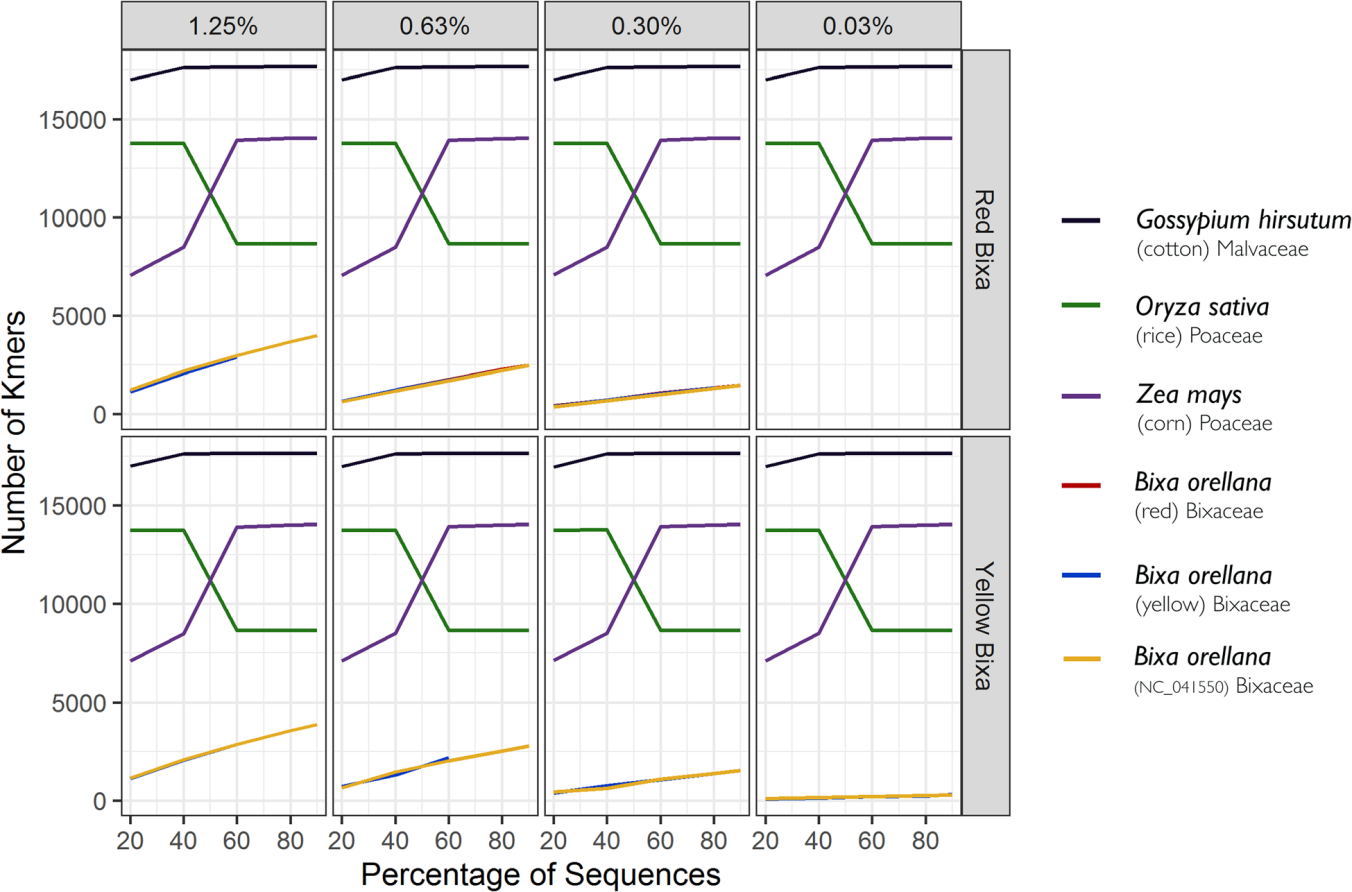
Percentages of sequences (out of 2.2 million; ie; 220,000 = 10%, etc.) on the x axis, and number of kmers that match the query taxa on the y axis. *Bixa* was successfully identified in a mixture of *Oryza sativa* 30%, *Zea mays* 30%, *Gossypium hirsutum* 30%, *Pseudomonas aeruginosa* 3.3%, *Salmonella enterica* 3.3%, and *Escherichia coli* 3.3%; at 5, 2.5, 1.25, 0.6, 0.3% and at some poorly supported levels of 0.03%, and was lost in all subsequent “dilutions”. Shown here is successful identification of *Bixa* at 1.25, 0.63, 0.3 and 0.03%. (There is almost perfect overlap of all three *Bixa* genomes so it appears as only one line)

We combined our two assembled *Bixa* chloroplast genomes with all the chloroplast genomes for Malvales found within the NCBI Refseq repository (Table S1). This combined reference sequence data set was then used to format a searchable kmer based reference database with the software Genome2-ID https://www.dna4tech.com/portal/genome2-id/. There were three reference *Bixa* chloroplast genomes available (the two presented here and NC_041550). We evaluated the accuracy of using whole chloroplast genomes by preparing three separate databases where all combinations of two *Bixa* chloroplasts genomes were included, and then used the whole genome shotgun/skim data from our two samples (SRR10320715 and SRR10320716) as well as data from available SRA accessions (SRR7941588 - SRR7941591) as input to test if the kmer software would correctly and unambiguously identify the raw data as that of *Bixa orellana*. The use of three databases allows us to avoid the circularity of testing the same data used in chloroplast assembly against its own assembled chloroplast genome (referred to as a ‘take-one-out’ strategy). We observed a 100% accuracy in assigning *Bixa* sequence data to *Bixa* reference chloroplast genomes (e.g. Figure 4). When *Bixa* sequence data was combined with other data, we again observed that *Bixa* could be found even when combined at a level of only 0.3% of all sequence data in a query (Fig. 4).

## Discussion

As we work towards a higher resolution understanding of the total ecology and biochemistry of food and the complex intersection it has with mammalian microbiota and states of health and disease, the organization of tools and methods to describe the full spectrum of ingredients in food and animal feeds is crucial. Depending on the question at hand, there are times when chemical approaches may be more appropriate and times when DNA based methods may be more relevant, and times when both should be used in unison. Either way, high quality reference databases are crucial to the accurate description of plant materials in foods.

DNA based methods are already firmly integrated into a wide array of continually modernizing authentication and quality assurance protocols. Recently, it has been shown that DNA plant barcodes (rbcL+matK) do not perform as well as full chloroplast genomes for closely related species [5, 6]. Work by Zhang et al. demonstrated that complete plastomes provided unambiguous resolution where official DNA barcodes failed to discriminate among closely related *Echinacea* species [1, 12]. Thus, use of entire genomes, plastomes and mitochondria, has become the gold standard to enable unambiguous discrimination between closely related species.

### Kmer based detection and phylogenetic inference

A next frontier for DNA based methods is the use of kmers generated from chloroplast genomes to create numerous specific targets that can detect the presence of specific species in complex mixtures using metagenomic and/or shotgun data sets of food and food ecologies. This approach was evaluated here for the identification of *Bixa orellana* from plant mixtures. The full genomes of *Bixa orellana* were sequenced, kmers were developed, and mixtures were queried against kmer formatted reference genome databases. *Bixa orellana* was consistently identified even when present at very small percentages of the total mixture with the lowest limit to exhibit reliable diagnostic capacity being 0.3% (Fig. 4). Thus, use of the entire chloroplast genome functioned as a genome scale DNA barcode and readily distinguished *Bixa*, even in admixture.

Use of complete chloroplast genomes can also provide reliable insight into phylogenetic relationships among groups. An increasing number of published studies have applied a combination of coding genes extracted from diverse chloroplast genomes for use in phylogenetic reconstruction [13, 14]. We demonstrated here that kmer based alignment free distance metrics, D2 and D2*, can be used to estimate genetic distances among a set of representative Malvales chloroplast genomes (Table S1). We observed with kmer distance metrics and traditional alignment based barcoding (and MLST) phylogenies that Bixaceae was sister to the Malvaceae.

### Biology and chemistry of Bixa oreallana

**Fig. 5:**
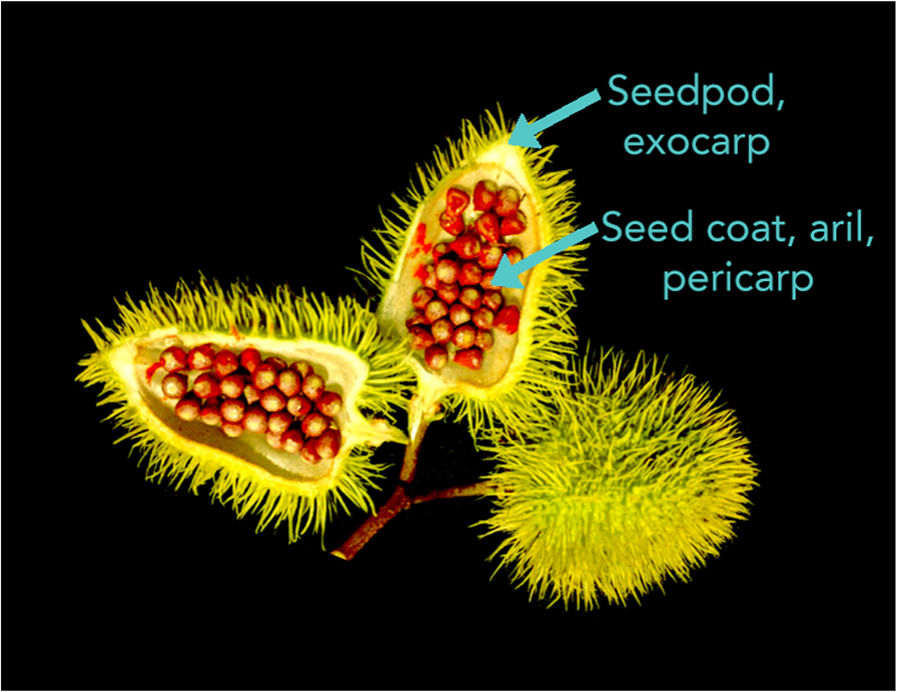
Seed pods and seed arils of *Bixa orellana*

*Bixa orellana* was identified for complete chloroplast sequencing because of its importance in many food and feed formulations. Arils of *Bixa* seeds produce bixin and norbixin apocarotenoids which impart bright red, orange, and yellow colors used in cheeses, butter, oils, margarine, ice-cream, candy, bakery products and rice [4]. The ‘presscake’ of the seedpod is also used as fodder for animals [4] and in poultry feed to provide rich shades of yellow and orange in egg yolks and chicken flesh. Leaves of *Bixa orellana* also have a long history of use in traditional medicine throughout the Americas [15–17] and extracts of the fruit and leaf have demonstrated antibiotic activity against *Staphylococcus aureus, Escherichia coli* and *Salmonella typhi*. Activity against eukaryotic parasites (as a vermifuge) has also been described [4]. The seedcoat (aril) (Fig. 5), of *Bixa orellana* is the part of the plant where bixin (the monomethyl ester of a dicarboxylic carotenoid (C_25_H_30_O_4_)), and norbixin ((C_24_H_26_O_4_), the saponified form of the same carotenoid) are produced [4].

Annatto carotenoids are known as ‘E160(b)’ in the European Union (EU) and use of annatto is permitted in a variety of food products. In the United States, while there has been limited reporting of potential allergens [18], annatto seed extracts are FDA-approved color additives for food, drugs, and cosmetics. Additionally, FDA had no questions regarding the conclusion that a preparation of annatto seed extract as a source of vitamin E in certain foods is generally recognized as safe (GRAS) [19, 20]. This formulation was focused on the tocotrienols and tocopherols present in the seeds [21]. The major pigment of the fruit is *cis*-bixin (~ 80%) with smaller amounts of other carotenoids. *Cis*-bixin is partially converted into the more stable *trans* isomer (which has the bright red color) upon heating which also produces a yellow byproduct. The quantity of trans isomer and yellow byproduct (red: yellow) balance is primarily influenced by temperature and duration of the heating processes [22].

Use of natural color from *Bixa orellana* in food and textile industries continues to grow in popularity as the use of similar red and yellow colors provided by synthetic dyes (especially azo dyes) are linked with environmental toxicity [23–26]. The International Union of Pure and Applied Chemistry (IUPAC) defines azo compounds as “derivative of diazene (diimide), HN=NH, wherein both hydrogens are substituted by hydrocarbyl group, e.g. PhN=NPh azobenzene or diphenyldiazene” [27]. These dyes are not typically removed in conventional waste water management and have been linked with toxicity to humans (cancers), aquatic organisms (fish and algae), as well as animals and microbes [23–26, 28]. For all of these reasons, the ability to identify this ingredient in food and feed preparations, either by chemical or molecular means, is of value for multiple reasons.

## Conclusions

The structure of the two chloroplast genomes presented here was effectively identical to that of the previously published chloroplast genome of *Bixa orellana* (NC_041550.1). Complete synteny was maintained, and the size and gene content of the inverted repeats was the same in all three accessions (Table 1). We found several potentially diagnostic insertion-deletion mutations that may differentiate red and yellow phenotypes – (subject of future work with increased numbers of individuals). Accession NC_041550.1 exhibited a 17 base gap at 58,299 in a intergenic spacer between *atp*B and *rbc*L relative to the other accessions, ‘red’ *Bixa* contained a 6 base gap at 75,537 in the intronic region of *clp*P, in addition to a snp at 86493. The number of genes total was consistent with that observed in other Malvales (Table 1) with the exception of *Heritiera fomes* which had an expanded inverted repeat which resulted in the duplication of several genes that are single copy in other Malvales. Additionally, we demonstrated that chloroplast genomes, used in conjunction with modern bioinformatic search tools (kmer based) can provide rapid and precise identification of *Bixa orellana* from mixtures of species for use with modernized Next Generation Sequencing (NGS) approaches to describe complex animal feeds and plant based food products with high accuracy.

## Methods

### Sampling, library preparation and sequencing

Individuals were collected from *Bixa orellana* accessions in the display/research garden of the Instituto de Medicina Tradicional de ES SALUD (IMET), (Iquitos, Peru) and the germplasm collection of *Bixa orellana* in the Facultad de Agronomía - Universidad Nacional de la Amazonía Peruana (UNAP). DNA extraction from leaves was performed using DNeasy Plant Mini Kit from Qiagen® according to the manufacturers specifications. DNA was prepared for sequencing using the Nextera™ DNA Flex Library Prep Kit according to the manufacturer’s specifications. Libraries were loaded onto an Illumina MiSeq using a V2 cartridge with read lengths set for (2 × 250).

### Sequence assembly

Following DNA sequencing, data were trimmed to remove adapters and indexes using BCL2fastq. Sequences were then paired and quality trimmed using Usearch11 [8]. Quality trimming parameters trimmed sequences to a median Q score of 25, with minimum length of 100 bp, and sequences below Q25 or 100 bp length discarded. Following read preparation, genome assembly followed the workflow described in [29] where reads are initially mapped to the nearest relative to enrich for chloroplast derived reads, and then denovo assembled to establish initial contigs, followed by subsequent subdivision and reassembly of contigs in an iterative fashion until the set of contigs can be combined to single complete chromosome. Completion of assembly was inferred by identifying inverted repeats and overlap between beginning and ending sequence reflecting assembly of a complete circle.

Paired, filtered reads were mapped to *Bixa orellana* (NC_041550) to enrich reads for chloroplast specific sequences using Geneious R11 (https://www.geneious.com) Mapped reads were then denovo assembled using SPAdes [30], with kmer sizes 21, 35 and 55. In parallel, all paired, filtered reads were denovo assembled with SPAdes to generate contigs independently of the reference genome to ensure all changes in structure and synteny could be identified. The denovo contigs of both assemblies were merged and assembled in Geneious R11. Assembled contigs were compared to the reference genome and gaps were filled by extracting sequences adjacent to the gaps and mapping the paired filtered reads to those sequences using Bowtie assembler [31] in a reference guided assembly. Contigs extended by Bowtie were then subsequently combined with previous contig sets and again compared to the reference genome. The assembled plastome was tested for the large inverted repeats, and the presence of both repeats was indicative of complete chromosome assembly. Raw sequence data were deposited in NCBI Sequence Read Archive (SRR10320715 and SRR10320716) as part of the FDA GenomeTrakrCP: chloroplast DNA for botanical product identification and for the genesis of the CVM Complete Chloroplast Animal Feed Database ([15], BioProject PRJNA325670).

### Sequence annotation and comparative gene content

Sequences were annotated with the Verdant online chloroplast assembly tool [32] (see: http://verdant.iplantcollaborative.org/plastidDB/). The existing reference for *Bixa*, NC_041550, was added to the Verdant database prior to upload and analysis of our sequences. The resulting gff files were downloaded and used to annotate the assembled *Bixa* chloroplast genomes (Fig. 2). Each CDS was extracted and translated to ensure correct open reading frames, and tRNA were extracted and assembled with homologous tRNA from the reference *Bixa* to ensure annotation captured the entire gene. Following evaluation and validation of all annotated genes, annotations were exported with the position, identity and phase of each gene. We then compared the number, synteny and organization of the set of genes relative to other chloroplast genomes within the Malvales (*Theobroma* and *Heritiera* (Malvaceae), *Vatica* (Dipterocarpaceae) and *Daphne* (Thymelaeceae)). Genes were classified based on the type of gene (CDS, tRNA, rRNA) as well as their organization within the chromosome (Large Single Copy (LSC), Inverted Repeat (IR), Small Single Copy (SSC)). The order of all genes was established and changes in the structural order of genes along the chromosome for all 5 chloroplasts was compared. Gain or loss of genes was denoted, and movement of genes among subunits of the genome (LSC, IR, SSC) based on expansion of the Inverted Repeat was recorded.

### Phylogenetic inference

Representative Chloroplast genome sequences for Malvales were downloaded from the GenBank RefSeq library for use in evaluation of the monophylly of the *Bixa orellana* genomes, as well as for inference of the relationship of Bixaceae to Malvales, and in particular with respect to its status as sister group to either Malvaceae or Thymelaeceae. The two Bixa assembled in this study in addition to the existing Bixa reference genome were included in a group of 37 additional Malvales chloroplast genomes (see Table S1) as well as *Arabidopsis thaliana* from the sister order Brassicales as an outgroup. The 41 chloroplast genomes were analyzed with the Café (accelerated alignment free sequence analysis [33] kmer distance program to infer genetic distance (using k = 8 in conjunction with the D2 genetic distance metric [34]. The resulting phylip formatted distance matrix was imported in PAUP [35] where *Arabidopsis thaliana* was set as the outgroup, and a NJ distance tree was computed. The same cadre of species was used with an extracted set of barcoding regions (matK and rbcL) and a 5 gene MLST schema comprised of rpoC1 (1–3116) rbcL (3117–4546) matK (4547–6154).

atpB (6155–7667) accD (7668–10,198) to generate additional phylogenic trees based on universal alignment. NJ distance trees were computed and phylograms for all three trees were visualized in Fig Tree v1.4.4. To share distance values, phylogenetic trees are shared in Supplementary Materials Fig. 1.

### Kmer identification from mixtures

*Genomes Oryza sativa* (DRR228665), *Zea mays* (SRR10812881), *Gossypium hirsutum* (SRR10081030), *Pseudomonas aeruginosa* (DRR215956), *Salmonella enterica* (SRR11851888), and *Escherichia coli* (SRR11851885) were downloaded from NCBI. Bacteria were added as a “contaminant” to test potential interference). All sequences were trimmed to 125 kb, and then combined in the following proportions; *Oryza sativa* (DRR228665) 30%, *Zea mays* (SRR10812881) 30%, *Gossypium hirsutum* (SRR10081030) 30%, *Pseudomonas aeruginosa* (DRR215956) 3.3%, *Salmonella enterica* (SRR11851888)3.3%, and *Escherichia coli* (SRR11851885) 3.3%. Mixed data was subsequently combined with different proportions of *Bixa orellana* plastome data and analyzed using Genome2-ID portal, a metagenomic software for plant taxonomic classification using kmers (DNA4 Technologies, LLC). To evaluate limit of detection, random selection of *Bixa* at 5, 2.5, 1.25, 0.6, 0.3, 0.15, 0.07, 0.03, 0.01%, were added to the mixture. Genome2-ID algorithms were run on mixed “dilutions” to identify, based on this approach, the point at which there was until no longer statistical support for identification of *Bixa* from the mixed sample.

## Supplementary information

**Additional file 1: Table S1.** Accession numbers, Family, Genus and species of all chloroplasts used for phylogenetic analyses.

**Additional file 2: Table S2.** Genes and gene synteny across Malvales.

**Additional file 3: Table S3.** Phylip formatted D2 distance matrix from kmer distances.

**Additional file 4: Supplementary Fig. 1**. Phylogenies of Bixaceae and Malvales species using A) Kmer distance based and B) 5 gene MLST universal alignment approaches.

## Data Availability

Raw sequence data were deposited in NCBI Sequence Read Archive (SRR10320715 and SRR10320716) as part of the FDA GenomeTrakrCP: chloroplast DNA for botanical product identification, BioProject PRJNA325670).

## Abbreviations

NGS: Next Generation Sequencing
LSC: Large Single Copy
IR: Inverted Repeat
SSC: Small Single Copy
CDS: CoDing Sequence
DNA: DeoxyriboNucleic Acid
NCBI: National Center for Biotechnology Information
Q score: Quality score
NARMS: National Antimicrobial Resistance Monitoring System
