# Supplementary figures and images for: Using full chloroplast genomes of ‘red’ and ‘yellow’ Bixa orellana (achiote) for kmer based identification and phylogenetic inference
Source: BMC Genomics. 2020 Aug 6;21:544. doi: 10.1186/s12864-020-06916-0 (PMC7430826; doi:10.1186/s12864-020-06916-0)

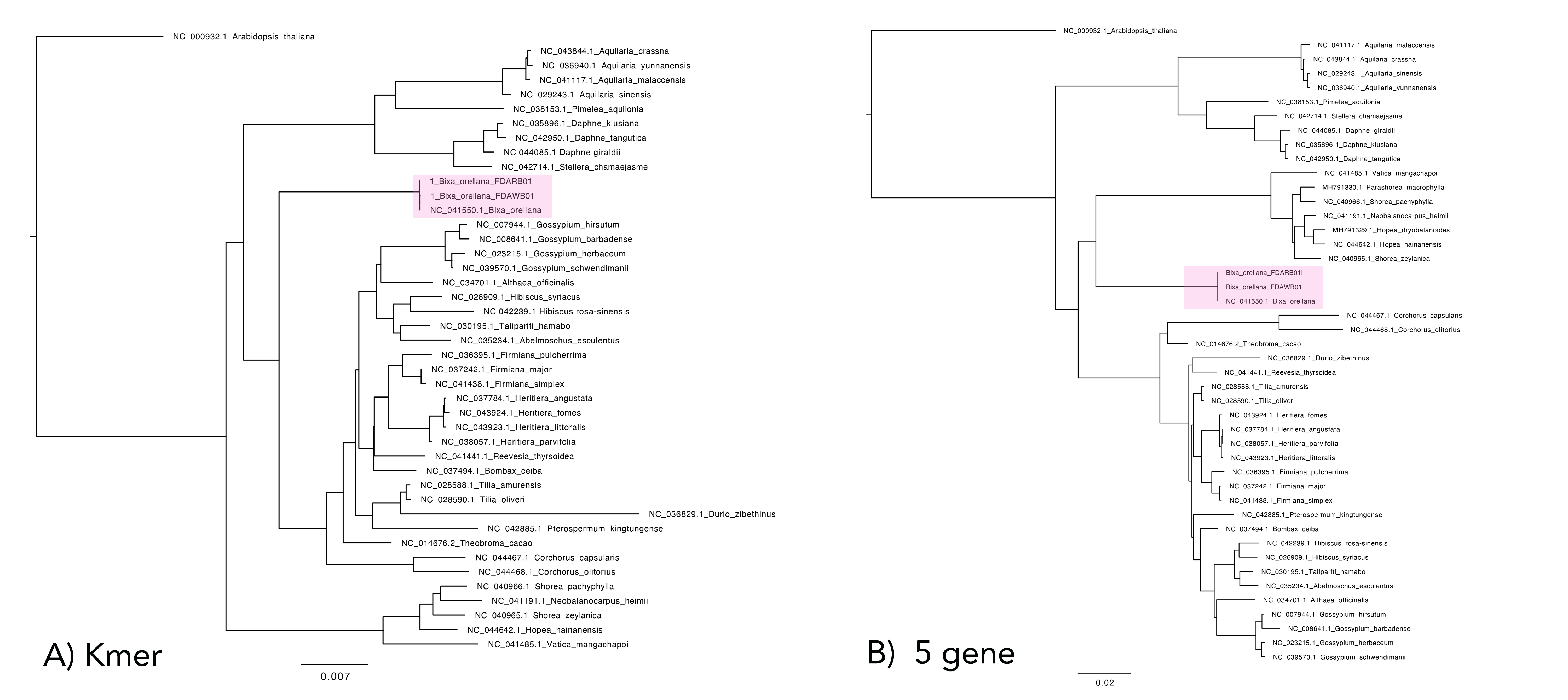

Supplement: Supplementary file 4 — Additional file 4: Supplementary Fig. 1. Phylogenies of Bixaceae and Malvales species using A) Kmer distance based and B) 5 gene MLST universal alignment approaches. [file 12864_2020_6916_MOESM4_ESM.jpg]
